# Comparison of Effects of Acellular Dermal Matrix and Latissimus Dorsi Muscle Flap on Radiation-induced Peri-implant Capsular Contracture in a Rabbit Model

**DOI:** 10.1055/a-2368-1813

**Published:** 2024-09-09

**Authors:** Suk Yoon Jang, Il Young Ahn, Tae Hui Bae, Shin Hyuk Kang, Soo Hyun Woo, Woo Ju Kim, Mi Kyung Kim, Chanutchamon Sutthiwanjampa, Han Koo Kim

**Affiliations:** 1 Department of Plastic and Reconstructive Surgery, Chung-Ang University, Graduate School of Medicine, Seoul, Republic of Korea; 2 Department of Plastic and Reconstructive Surgery, Chung-Ang University Hospital, Seoul, Republic of Korea; 3 Department of Plastic and Reconstructive Surgery, Chung-Ang University Gwangmyeong Hospital, Gyeonggi-do, Republic of Korea; 4 Department of Pathology, Chung-Ang University Hospital, Seoul, Republic of Korea; 5 Department of Integrative Engineering, Chung-Ang University, Seoul, Republic of Korea

**Keywords:** silicone breast implant, acellular dermal matrix, latissimus dorsi muscle flap, radiation-induced capsular contracture

## Abstract

**Background** Capsular contracture of breast implants is a major complication in breast surgery. Clinically, covering a breast implant with acellular dermal matrix (ADM) or autologous tissue is considered to be the most effective technique to prevent capsular contracture. This study was designed to compare the protective effects of ADM and latissimus dorsi (LD) muscle flap placement on capsular contracture by increasing the rate of capsular contracture through controlled radiation exposure in a rabbit model.

**Methods** Twenty New Zealand white rabbits were divided into three groups. After the implant was placed beneath the pectoralis major muscle, the lateral third of the implant was left exposed in the control group ( *n* = 6). In the ADM group ( *n* = 7), the exposed implant was covered with AlloDerm. In the LD flap group ( *n* = 7), the exposed implant was covered with a pedicled LD muscle flap. All groups were irradiated 3 weeks after implant insertion. After 6 months, peri-implant tissues were harvested and analyzed.

**Results** ADM showed markedly lower myofibroblast activity than the LD flap. However, transforming growth factor-β1 levels and the activity of collagen types I and III produced in fibroblasts were significantly lower in the ADM group than in the LD flap group.

**Conclusion** Based on the findings of our rabbit experiments, ADM is expected to have a comparative advantage in reducing the risk of capsular contracture compared to the LD flap.

## Introduction

Capsular contracture is a significant postoperative complication following breast implantation, impacting both cosmetic and reconstructive breast surgeries. This condition, characterized by fibroproliferative changes, leads to considerable morbidity in implant-based breast reconstruction. To better understand and address this issue, a variety of animal models have been employed to simulate capsular contracture. 1 2 3 Notably, rabbit models have been less explored in this context. From the study of Adams et al, 1 it has been shown that pathological capsular contraction in humans can be similarly induced in rabbits. Although it exists, there are not many studies comparing inducing capsular contracture through controlled radiation exposure on rabbits. 2 3 4

In general, breast implant surgery is the first choice for breast reconstruction after mastectomy. It has been reported that radiation therapy after the insertion of a breast implant increases the risk of complications, such as capsular contracture, thinning of the skin, and extrusion of implants. Therefore, there is increasing interest in methods to reduce the risk of radiation-induced capsular contracture during breast reconstruction using breast implants.

Various methods have been introduced to reduce radiation-induced capsular fibrosis around silicone implants by using drugs, wrapping the implant with synthetic materials, and attempting a novel surgical method.

In one animal study, it was observed that 3-hydroxy-3-methylglutaryl coenzyme A reductase inhibitors such as simvastatin were effective in reducing radiation-induced capsular contracture around silicone implants. 5 It has been reported that treatment with pentoxifylline and vitamin E can prevent serious radiation-induced capsular contraction and breast implant loss. 6 Furthermore, many studies have evaluated the prevention of this complication using synthetic materials. Synthetic meshes are flexible sheets and play a similar role to biological matrices. These are classified as short-term resorbable meshes made of polyglycolic acid (Dexon) and polyglactin (Vicryl), long-term resorbable meshes made of silk protein (SERI surgical scaffold), or poly-4-hydroxybutyrate polymer (Galatea scaffold), and nonabsorbable meshes composed of polypropylene and polyester (Mersilene). However, few data are available. 7 There have also been efforts to prevent radiation-induced capsular contracture using an autologous fat graft. 8

Clinically, a single-stage direct-to-implant (DTI) breast reconstruction offers the advantages of a short operative time and lack of donor site morbidity. In the past, DTI breast reconstruction was abandoned because of possible complications such as implant malposition and contracture. However, acellular dermal matrix (ADM) offers a solution to these problems. The introduction of ADM made possible a method called dual plane which covers implant with partial subpectoral pocket and ADM on inferior pectoralis major muscle extending to the lateral mammary fold. 9 For this reason, DTI breast reconstruction using ADM on the dual plane has become more favorable in recent years over two-staged reconstruction. 10 11

DTI breast reconstruction with ADM is attractive to both patients and surgeons avoiding additional surgery. However, it may be difficult to use ADM in some cases because of cost issues or because patients decline to have ADM used for religious reasons. Additionally, ADM is still associated with a high possibility of complications including seroma and infection. 11 An alternative method in this situation is to cover the prosthesis with muscles. Several studies have reported the effectiveness of latissimus dorsi (LD) flap with implant breast reconstruction on aesthetic results and cost-effectiveness. 12 13 However, there are only a few data available on the comparison of ADM and muscle coverage on dual-plane. Therefore, this experiment was designed to compare the protective effect of ADM and an LD muscle flap against capsular contracture by inducing capsular contracture through controlled radiation exposure using a rabbit model.

## Methods

### Study Animals

Twenty 8-week-old, female, New Zealand white rabbits weighing 2.5 to 2.8 kg were used in this study. Pairs of rabbits were accommodated in individual cages with unrestricted access to nourishment and hydration, along with adherence to a consistent 12-hour light–dark cycle. The experiment was conducted at Chung-Ang University's Animal Research Laboratory and was approved by the Animal Review Committee of Chung-Ang University Hospital (approval number 2017-00019).

### Implant

**Fig. 1:**
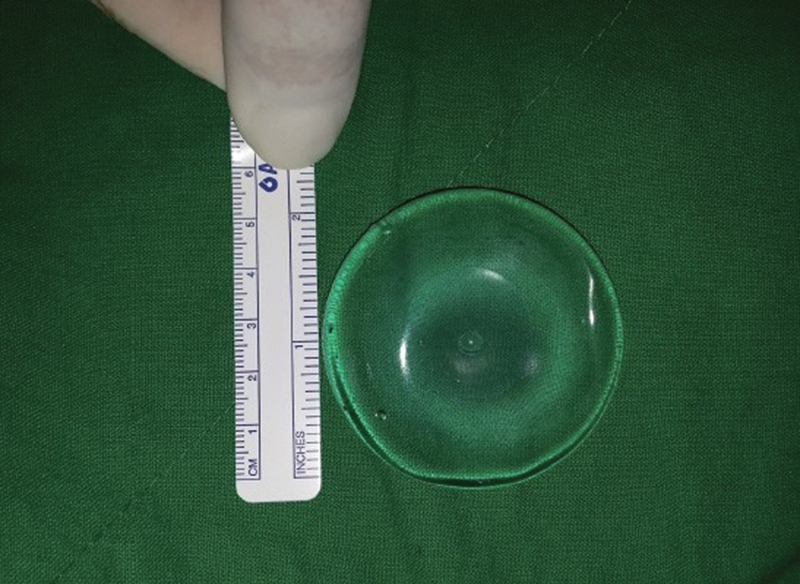
Overview image of prosthesis. A 5 cm × 5 cm × 1 cm sized smooth surface, round, cohesive gel implant (Hans Biomed Co., Seoul, Korea) was prepared for dual-plane insertion on a rabbit model.

Smooth, round-type, cohesive gel implants measuring 5 × 5 × 1 cm were manufactured in the same way as the product used in actual breast augmentation (Hans Biomed Corp., Seoul, Korea). To avoid infection, ethylene oxide sterilization was performed before implantation ( Fig. 1 ).

### Study Procedures

**Fig. 2:**
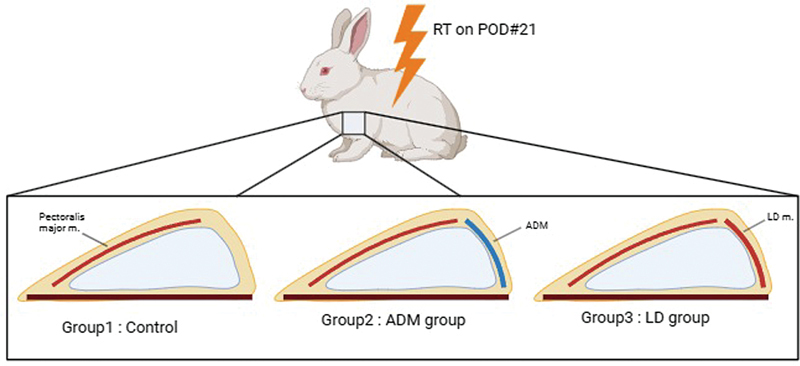
Overall schematic diagram of implant insertion. Each group of rabbits were divided into control, ADM, and LD groups. Every dorsal aspect of implant were covered with pectoralis major muscles. Each of lateral aspect of implant were not covered in control group, but covered with ADM and LD muscle in ADM group and LD group, respectively. ADM, acellular dermal matrix; LD, latissimus dorsi.

**Fig. 3:**
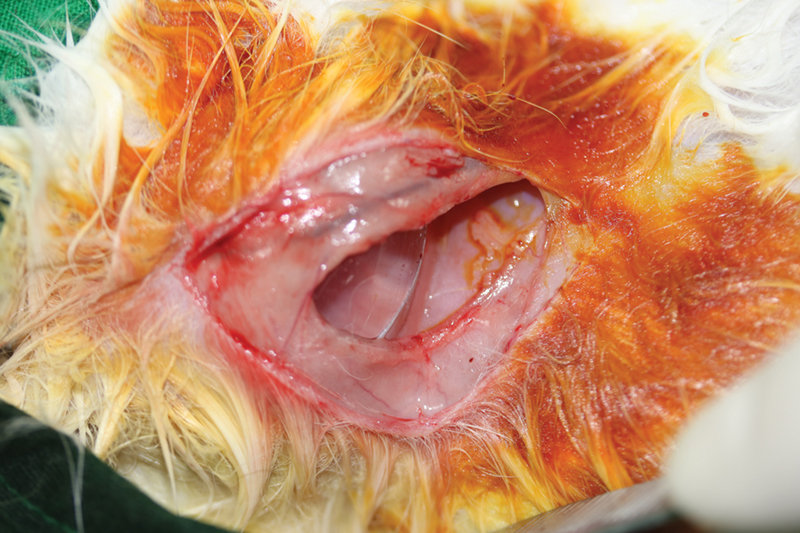
Operative view of the control group. The implant was inserted in the subpectoral area and covered with pectoralis muscle on dorsal part. One-third of exposed area (lateral part) was not covered with pectoralis muscle.

**Fig. 4:**
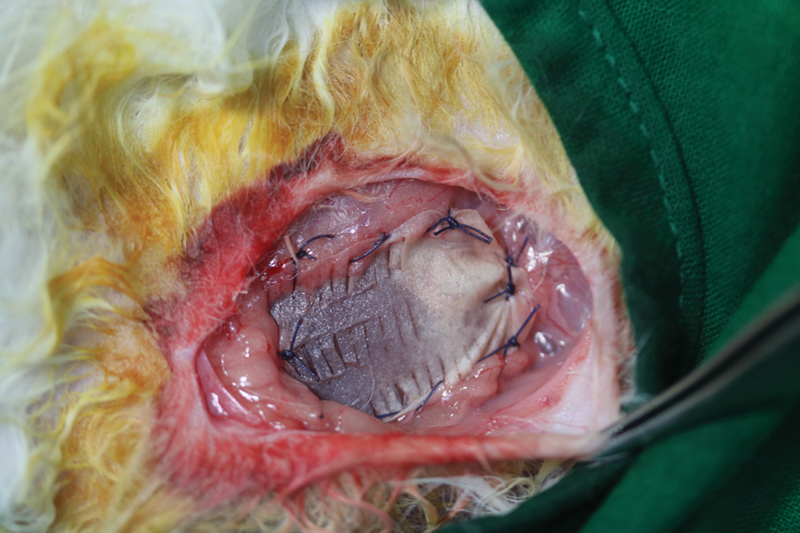
Operative view of the ADM group. The implant was inserted in the subpectoral area and covered with pectoralis muscle on dorsal part. One-third of exposed area (lateral part) was covered by AlloDerm. ADM, acellular dermal matrix.

**Fig. 5:**
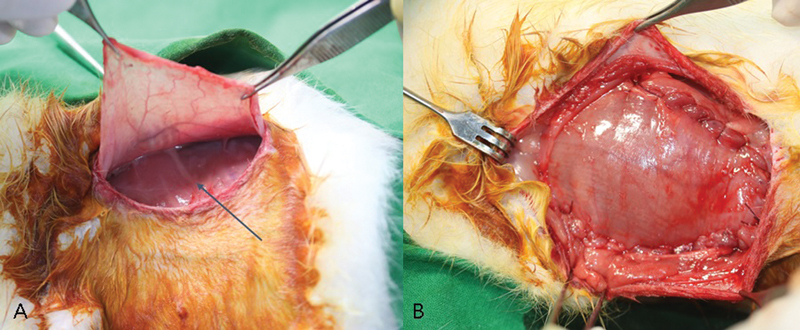
Operative view of the LD flap group. ( **A** ) LD muscle flap elevation was done and pedicle of the flap can be seen (arrow). ( **B** ) The implant was inserted in the subpectoral area and covered with pectoralis muscle on dorsal part. One-third of exposed area (lateral part) was covered by pedicled LD flap. LD, latissimus dorsi.

Twenty rabbits were divided into three groups according to the following criteria: control group ( *n* = 6), ADM group ( *n* = 7), and LD muscle group ( *n* = 7; Fig. 2 ). Anesthesia was induced using a 10 mg/kg tiletamine–zolazepam mixture (Zoletil, Virbac Korea, Korea) and 2 mg/kg xylazine hydrochloride (Rompun, Bayer Korea, Korea) via intramuscular injection. Preoperative preparations included shaving and cleaning the left axilla of each animal using 10% povidone-iodine. A 5-cm incision was made in the left armpit, the plane beneath the pectoralis major muscle was approached, and dissection was performed to the midline to create a pocket for the implant. After the implant was placed beneath the pectoralis major muscle, the lateral one-third of the implant was left exposed in the control group ( *n* = 6; Fig. 3 ). In the ADM group ( *n* = 7), the exposed implant was covered with 9 to 13 × 10 ^−3^ inch thick AlloDerm (LifeCell Corp., United States; Fig. 4 ) In the LD flap group ( *n* = 7), the exposed implant was covered with a pedicled LD muscle flap ( Fig. 5 ). After surgery, for each rabbit, 20 mg/kg of cefazolin (Cefazolin; Chong Kun Dang Pharmaceutical Corp, Korea) was injected intramuscularly to prevent postoperative infection. All experiments were performed in sterilized and stable conditions.

### Radiation

Three weeks after implant insertion, each group was planned to undergo a single dose of irradiation to induce capsular contracture. On postoperative day 21, a single dose of 25 Gy from cobalt-60 (Co-60) gamma radiation was administered. To safeguard the internal organs from exposure and focus the radiation solely on the implant area, the rabbits received anesthesia (10 mg/kg tiletamine–zolazepam mixture [Zoletil, Virbac Korea, Korea] and 2 mg/kg xylazine hydrochloride [Rompun, Bayer Korea, Korea] via intramuscular injection) with the identical agents used during the implant procedure.

### Tissue Harvesting and Evaluation

**Fig. 6:**
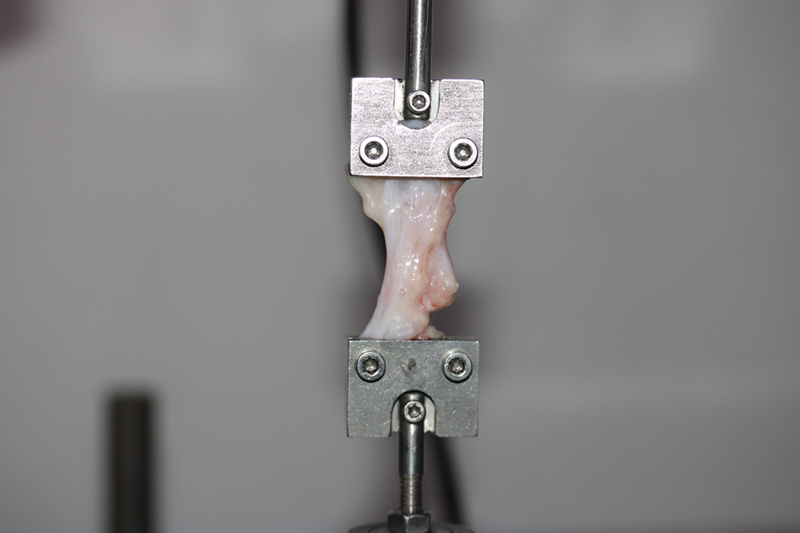
Tensile strength measurement. Tensile strength was measured with a tensiometer. The tensile strength of the capsule was measured from tissue directly above the ADM or LD flap or control (lateral parts). Each of harvested tissue was prepared into 1 × 3 cm sized piece of capsule for measuring capsule tensile strength and was measured with a tensiometer. ADM, acellular dermal matrix; LD, latissimus dorsi.

Six months postoperatively, the animals were euthanized by carbon dioxide intoxication, and the implants with the peri-implant capsule were harvested. Animals were sacrificed with minimal pain in a carbon dioxide chamber. The tissue was extensively excised and all tissues with a capsule formed around the implant were collected. For precise thickness measurement, tissues were collected directly above the ADM or LD flap (lateral parts) which were covered with ADM/LD flap or control not pectoralis muscles. After precise tissue separation from the implants, each of the harvested tissue from the control group, ADM group, and LD group was prepared into 1 × 3 cm sized piece of capsule for measuring capsule tensile strength and was measured with a tensiometer ( Fig. 6 ). After measuring physical properties, each tissue from the dorsal parts (covered by the pectoralis muscle) and lateral parts (covered by ADM or an LD flap/control) of the capsule were separately collected for quantitative immunohistochemical stain analysis. Each of the collected tissues from the dorsal and lateral parts of the control, ADM, and LD groups was sliced into three pieces for quantitative immunohistochemical stain analysis. The tissues were fixed in 10% formalin solution for histopathologic evaluation for more than 24 hours. For quantitative reverse transcription polymerase chain reaction (RT-qPCR) analysis, each tissue from lateral parts (covered by ADM or an LD flap/control) of the capsule was selected. Tissues were snap-frozen in liquid nitrogen-cooled isopentane and stored at −80 °C for RT-qPCR analysis.

### Pathologic Evaluation

For robust statistical validation, triplicate specimens from both the dorsal and lateral aspects of each capsule were harvested and sliced into three pieces for precise analysis. Specimens were stained for hematoxylin and eosin (H&E) staining (Vector Laboratories, Burlingame, CA), Masson's trichrome (MT) staining (Sigma–Aldrich), α-SMA (Abcam, Cambridge, MA), transforming growth factor (TGF)-β1 (TGF-β1; Abcam, Cambridge, MA), and TGF-β2 (Abcam, Cambridge, MA) for immunohistochemical analysis. Counterstaining was done with hematoxylin. H&E staining was performed to measure the thickness of the capsule, and measurements were made using ImageJ software (National Institutes of Health, Bethesda, MD). Capsules with the least thickness were analyzed from the H&E images. MT staining was used to identify collagen fiber in the capsule. Quantitative analysis was performed for α-SMA, TGF-β1, and TGF-β2. Quantitative analysis was recorded based on the following percentages of expression level: 0% as ±, 0 to 30% as 1 + , 30 to 70% as 2 + , and 70% and above as 3 + . The samples of dorsal and lateral parts were reviewed by a blinded pathologist.

### Quantitative Reverse Transcription Polymerase Chain Reaction

RT-qPCR analysis was performed to evaluate α-SMA, TGF-β1, SMAD3, and Collagen type I and III. Total RNA from the harvested tissues from lateral parts (covered by ADM or an LD flap/control) of the capsule was extracted using an easy-spin RNA extraction kit (iNtRON, Korea) according to the manufacturer's instructions. The extracted mRNA was converted to complementary DNA using Rocketscript RT premix (Bioneer, Korea). RT-qPCR was performed using SYBR Green Master Mix (Greenstar qPCR Master, Bioneer, Korea) and Rotor-Gene Q (Qiagen). The expression level of each gene was calculated using the cycle threshold (CT) method. The ΔCT value of each gene was calculated by subtracting the CT value of β-actin from the respective gene-specific CT values. To assess relative gene expression (ΔΔCT), the ΔCT value of the experimental group was subtracted from the ΔCT value of the control group; then, the fold-change was calculated as 2 ^−ΔΔCT^ .

### Statistical Analysis

Data are expressed as mean ± standard deviation, and statistical analysis was performed using Sigma Plot (v. 12.0; Systat Software Inc., Point Richmond, CA). Due to the small size of the samples with only six to seven animals in each group, a nonparametric analysis was selected to compare the mean values of each group. Each immunohistochemical evaluation was scored on a point-based system (0 points for ±, 1 point for 1 + , 2 points for 2 + , and 3 points for 3 + ). Statistical analyses were performed using the Kruskal–Wallis test to compare the three groups. The results were evaluated at a significance level of *p* < 0.05.

## Results

### Animal Survival

Two rabbits died a week after radiation in the ADM group and LD flap groups. The other 18 rabbits survived until 6 months after surgery. There were no surgical complications such as implant exposure or wound infection.

### Capsule Thickness and Tensile Strength

**Fig. 7:**
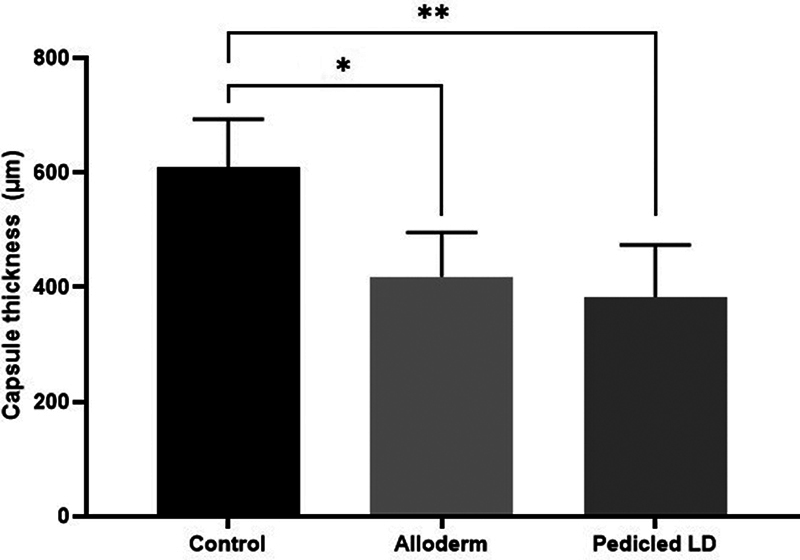
Bar graphs showing capsule thickness. The thickness of the capsule was measured from tissue directly above the ADM or LD flap or control: control group, 609.2 ± 76.60 μm; ADM group, 418.0 ± 67.72 μm; LD group, 382.83 ± 78.95 μm. Each of the ADM group and LD group showed statistically significant differences when compared with the control group. ADM, acellular dermal matrix; LD, latissimus dorsi. * *P* ≤ 0.05. ** *P* ≤ 0.01.

**Fig. 8:**
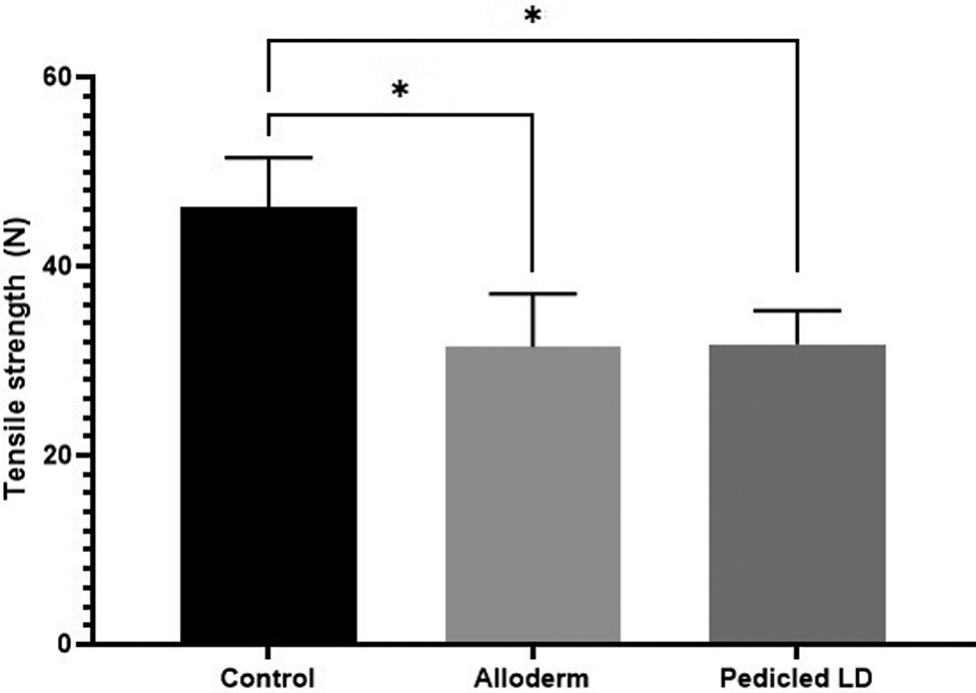
Bar graphs showing tensile strength. The tensile strength of the capsule was measured from tissue directly above the ADM or LD flap or control: control group, 46.2 ± 4.84 N; ADM group, 31.5 ± 5.12 N; LD group, 31.8 ± 3.20 N. Each of ADM group and LD group showed statistically significant differences when compared with the control group. ADM, acellular dermal matrix; LD, latissimus dorsi. * *P* ≤ 0.05.

The mean capsular thickness measurements were 609.2± 76.60 μm in the control group, 418.0 ± 67.72 μm in the ADM group, and 382.83 ± 78.95 μm in the LD flap group. The ADM group and LD flap group showed a significantly thinner capsule than the control group ( Fig. 7 ). The mean capsular tensile strength values were 46.2 ± 4.84 N in the control group, 31.5 ± 5.12 N in the ADM group, and 31.8 ± 3.20 N in the LD flap group. The ADM and LD flap groups exhibited significantly less tensile strength than the control group, which was similar in terms of capsule thickness ( Fig. 8 ).

### Pathologic Evaluation

**Fig. 9:**
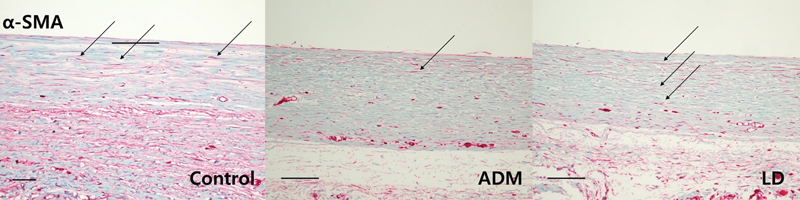
Immunohistochemistry of myofibroblasts (α-SMA). For quantitative immunohistochemical stain analysis of α-SMA, tissues were harvested from dorsal and lateral parts of control, ADM, and LD groups. The following pathology pictures are all tissues collected from the lateral side which is directly above the ADM or LD flap or control. Each arrow indicates an expression of α-SMA on each group (scale bar, 200 μm). ADM, acellular dermal matrix; LD, latissimus dorsi.

Immunohistochemistry for myofibroblasts, TGF-β1, and TGF-β2, and histologic sections of collagen fibers showed significant differences between the control group and both experimental groups ( Figs. 9 10 11 12 ). On statistical comparison of results of immunohistochemical evaluation scored on a point-based system, the ADM group showed significantly lower infiltration of α-SMA, TGF-β1, and TGF-β2 than the control group. Also, the LD group showed significantly lower values on α-SMA and TGF-β2 than the control group, but not in TGF-β1 ( Table 1 ).

**Table 1:** Statistical comparison of the results of immunohistochemical evaluation scored on a point-based system

Immunohistochemical stain	Control ( *n* = 3 × 6 = 18)	ADM ( *n* = 3 × 6 = 18)	LD ( *n* = 3 × 6 = 18)	*p* -Value
SMA				
Dorsal	2.056 ± 0.539	0.778 ± 0.428	1.000 ± 0.485	Control-ADM: 0.000 ^a^
				Control-LD: 0.000 ^a^
				ADM-LD: 0.999
Lateral	2.167 ± 0.618	1.222 ± 0.428	1.444 ± 0.511	Control-ADM: 0.000 ^a^
				Control-LD: 0.005 ^a^
				ADM-LD: 0.747
TGF-β1				
Dorsal	2.334 ± 0.485	0.889 ± 0.583	1.167 ± 0.618	Control-ADM: 0.000 ^a^
				Control-LD: 0.000 ^a^
				ADM-LD: 0.894
Lateral	2.111 ± 0.583	1.176 ± 0.529	1.556 ± 0.511	Control-ADM: 0.000 ^a^
				Control-LD: 0.051
				ADM-LD: 0.148
TGF-β2				
Dorsal	2.389 ± 0.502	1.111 ± 0.583	1.500 ± 0.514	Control-ADM: 0.000 ^a^
				Control-LD: 0.001 ^a^
				ADM-LD: 0.359
Lateral	2.222 ± 0.548	1.222 ± 0.548	1.611 ± 0.502	Control-ADM: 0.000 ^a^
				Control-LD: 0.022 ^a^
				ADM-LD: 0.207

Abbreviations: ADM, acellular dermal matrix; LD, latissimus dorsi; TGF-β1/2, transforming growth factor-β1/2.

Quantitative analysis was performed for α-SMA, TGF-β1, and TGF-β2. For robust statistical validation, triplicate specimens from both the dorsal and lateral aspects of each capsule were harvested and sliced into three pieces for precise analysis. Quantitative analysis was recorded based on the following percentages of expression level: 0% as ±, 0 to 30% as 1 + , 30 to 70% as 2 + , and 70% and above as 3 + . Each sample of dorsal and lateral parts was reviewed by a blinded pathologist. The following table displays the average of the obtained values, and compared the statistical significance between each group.

**Fig. 10:**
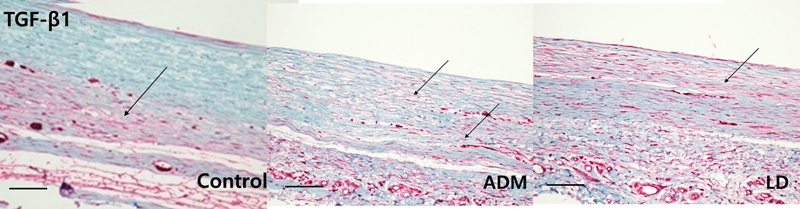
Immunohistochemistry of TGF-β1. For quantitative immunohistochemical stain analysis of TGF-β1, tissues were harvested from dorsal and lateral parts of the control, ADM, and LD groups. The following pathology pictures are all tissues collected from the lateral side which is directly above the ADM or LD flap or control. Each arrow indicates an expression of TGF-β1 on each group (scale bar, 200 μm). ADM, acellular dermal matrix; TGF-β1, transforming growth factor-β1.

**Fig. 11:**
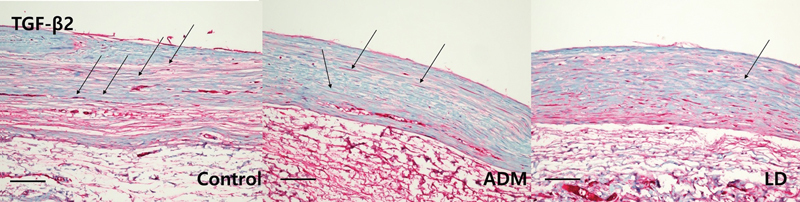
Immunohistochemistry of TGF-β2. For quantitative immunohistochemical staining analysis of TGF-β2, tissues were harvested from dorsal and lateral parts of the control, ADM, and LD groups. The following pathology pictures are all tissues collected from the lateral side which is directly above the ADM or LD flap or control. Each arrow indicates and expression of TGF-β2 on each group (scale bar, 200 μm). ADM, acellular dermal matrix; TGF-β2, transforming growth factor-β2.

**Fig. 12:**
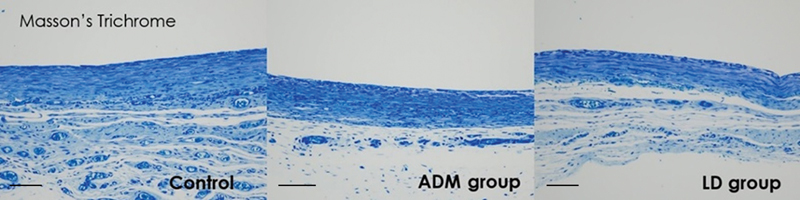
Histologic sections of collagen fibers. For histologic analysis of collagen fibers, Masson's Trichrome staining was done on harvested tissue. The following pathology pictures are all tissues collected from the lateral side which is directly above the ADM or LD flap or control (scale bar, 400 μm). ADM, acellular dermal matrix; LD, latissimus dorsi.

In the control group, the mean myofibroblast ratio on the dorsal aspect was 2.056 ± 0.539, and that on the lateral aspect was 2.167 ± 0.618. The TGF-β1 ratio on the dorsal aspect was 2.334 ± 0.485, and that on the lateral aspect was 2.111 ± 0.583. The TGF-β2 ratio on the dorsal aspect was 2.389 ± 0.502, and that on the lateral aspect was 2.222 ± 0.548. In the ADM group, the mean myofibroblast ratio on the dorsal aspect was 0.778 ± 0.428, and that on the lateral aspect was 1.222 ± 0.428. The TGF-β1 ratio on the dorsal aspect was 0.889 ± 0.583, and that on the lateral aspect was 1.176 ± 0.529. The TGF-β2 ratio on the dorsal aspect was 1.111 ± 0.583, and that on the lateral aspect was 1.222 ± 0.548. In the LD flap group, the mean myofibroblast ratio on the dorsal aspect was 1.000 ± 0.485, and that on the lateral aspects was 1.444 ± 0.511. The TGF-β1 ratio on the dorsal aspect was 1.167 ± 0.618, and that on the lateral aspect was 1.556 ± 0.511. The TGF-β2 ratio on the dorsal aspect was 1.500 ± 0.514, and that on the lateral aspect was 1.611 ± 0.502. Overall, there was significantly lower infiltration of α-SMA and TGF-β2 in both the ADM group and the LD group than the control group, but on TGF-β1, only the ADM group showed a significant difference. There were no significant differences between the ADM group and the LD flap group ( Table 1 ).

### Myofibroblasts (α-SMA) and Transforming Growth Factor-β1

**Fig. 13:**
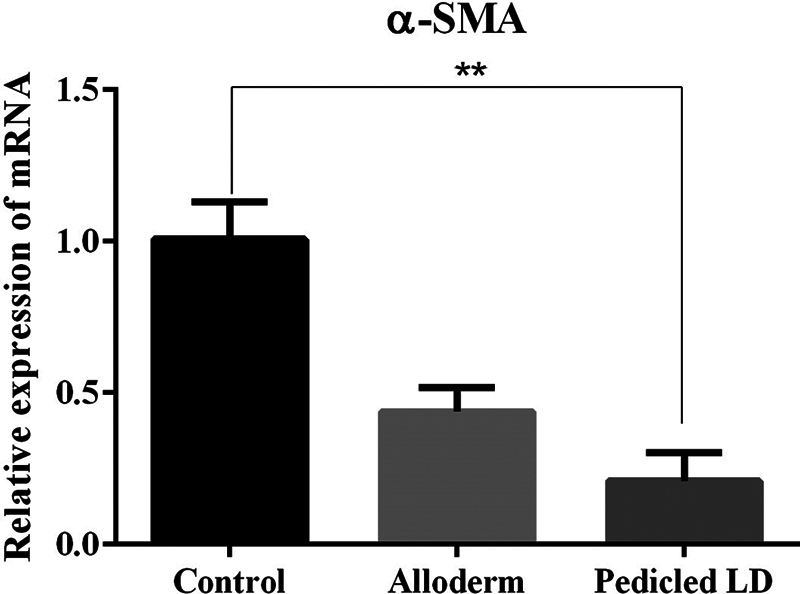
Bar graphs showing quantification of α-SMA using RT-qPCR. mRNA ratio expression of α-SMA from tissue directly above the ADM or LD flap or control: control group, 1.0062 ± 0.1229; ADM group, 0.4375 ± 0.0805; LD flap group, 0.2093 ± 0.0937. Only the comparison between the control group and the LD group a showed statistically significant difference. ADM, acellular dermal matrix; LD, latissimus dorsi; RT-qPCR, quantitative reverse transcription polymerase chain reaction. ** *P* ≤ 0.01.

To examine the myofibroblast content in the capsules, the α-SMA and TGF-β1 expression on the seventh capsule was detected by mRNA expression quantified by RT-qPCR. On α-SMA, significantly lower levels were detected in the LD group than in the control group. However, there was no significance in comparison between ADM group and control group or LD group (mRNA ratio expression of α-SMA: control group, 1.0062 ± 0.1229; ADM group, 0.4375 ± 0.0805; LD flap group, 0.2093 ± 0.0937; Fig. 13 ).

**Fig. 14:**
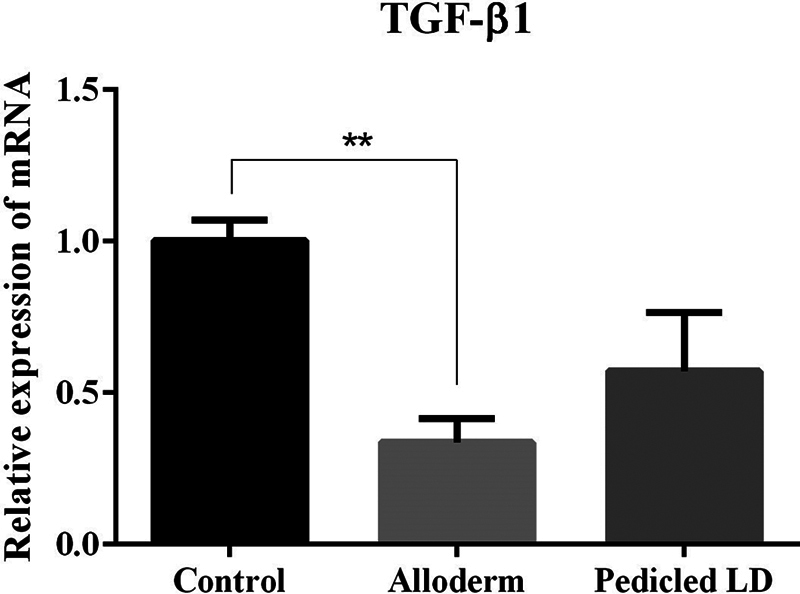
Bar graphs showing quantification of TGF-β1 using RT-qPCR. mRNA ratio expression of TGF-β1 from tissue directly above the ADM or LD flap or control: control group, 1.0016 ± 0.065; ADM group, 0.3364 ± 0.0938; LD flap group, 0.5717 ± 0.1942. Only the comparison between control group and ADM group showed a statistically significant difference. ADM, acellular dermal matrix; LD, latissimus dorsi; RT-qPCR, quantitative reverse transcription polymerase chain reaction; TGF-β1, transforming growth factor-β1. ** *P* ≤ 0.01.

On TGF-β1 expression, the ADM group showed significantly lower levels than the control group. There was no significance between LD group and control group or ADM group (mRNA ratio expression of TGF-β1: control group, 1.0016 ± 0.065; ADM group, 0.3364 ± 0.0938; LD flap group, 0.5717 ± 0.1942; Fig. 14 ).

### SMAD3

**Fig. 15:**
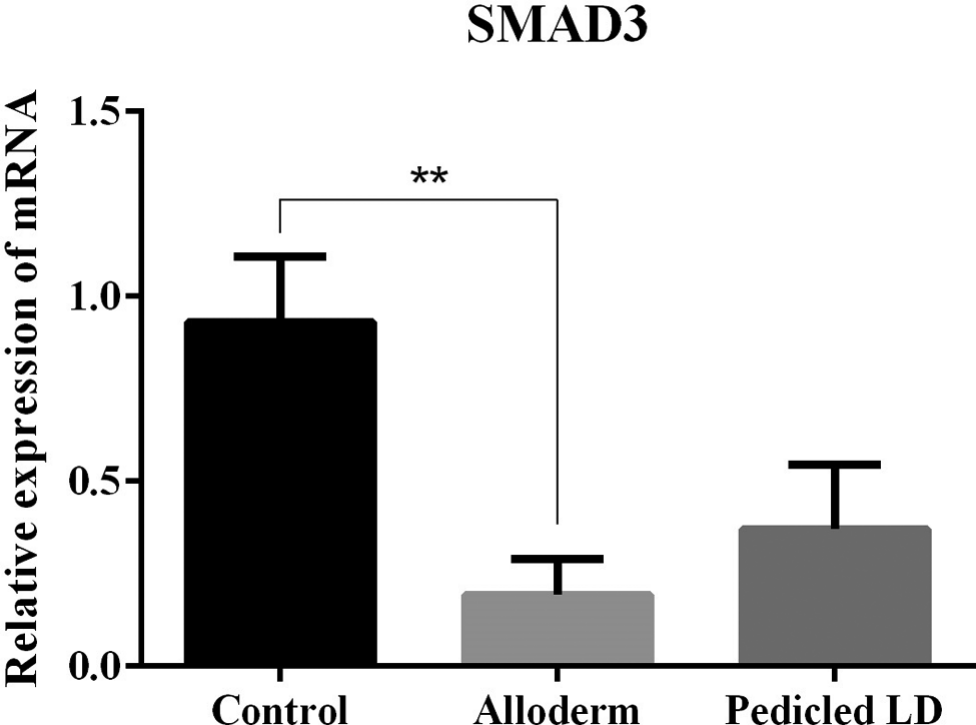
Bar graphs showing quantification of SMAD3 using RT-qPCR. mRNA ratio expression of SMAD3 from tissue directly above the ADM or LD flap or control: control group, 0.9306 ± 0.176; ADM group, 0.1926 ± 0.098; LD flap group, 0.3703 ± 0.174. Only the comparison between control group and ADM group showed a statistically significant difference. ADM, acellular dermal matrix; LD, latissimus dorsi; RT-qPCR, quantitative reverse transcription polymerase chain reaction. ** *P* ≤ 0.01.

SMAD3, which is associated with the TGF-β signaling mechanism, was detected by mRNA expression quantified by RT-qPCR. In comparison between the value of SMAD3, the ADM group showed significantly lower levels than the control group. There was no significant difference between the LD group and the control group or ADM group (mRNA ratio expression of SMAD3: control group, 0.9306 ± 0.176; ADM group, 0.1926 ± 0.098; LD flap group, 0.3703 ± 0.174; Fig. 15 ).

### Collagen Type I and III

**Fig. 16:**
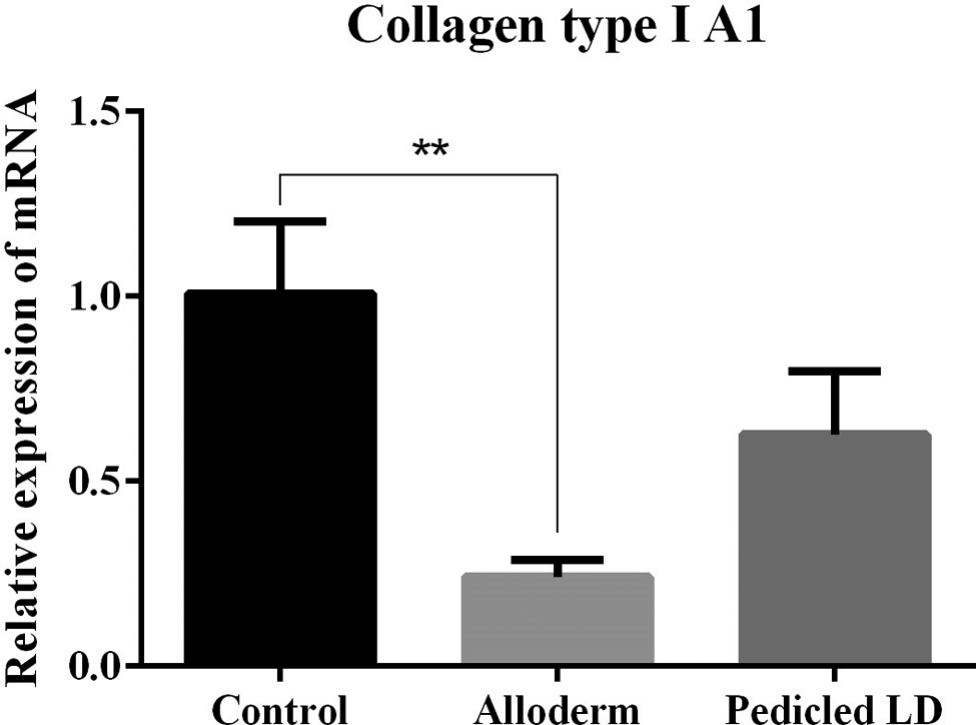
Bar graphs showing quantification of collagen type I using RT-qPCR. mRNA ratio expression of collagen type I from tissue directly above the ADM or LD flap or control: control group, 1.0084 ± 0.194; ADM group, 0.2418 ± 0.0463; LD flap group, 0.6265 ± 0.1708. Only the comparison between control group and ADM group showed a statistically significant difference. ADM, acellular dermal matrix; LD, latissimus dorsi; RT-qPCR, quantitative reverse transcription polymerase chain reaction. ** *P* ≤ 0.01.

**Fig. 17:**
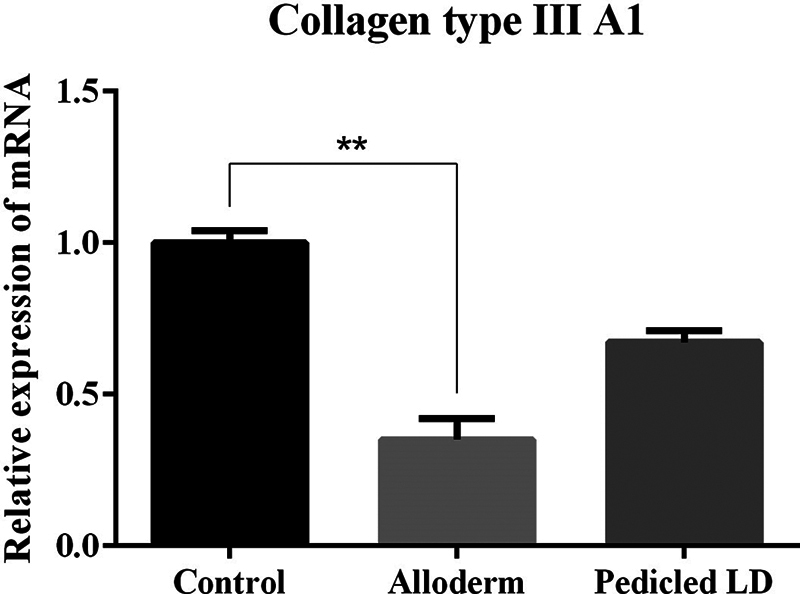
Bar graphs showing quantification of collagen type III using RT-qPCR. mRNA ratio expression of collagen type III from tissue directly above the ADM or LD flap or control: control group, 1.0006 ± 0.0382; ADM group, 0.3503 ± 0.0693; LD flap group, 0.6717 ± 0.0382. Only the comparison between control group and ADM group a showed statistically significant difference. ADM, acellular dermal matrix; LD, latissimus dorsi; RT-qPCR, quantitative reverse transcription polymerase chain reaction. ** *P* ≤ 0.01.

To examine representative collagen types known to be associated with fibrosis, collagen types I and III on the capsules were detected by mRNA expression quantified by RT-qPCR. Significantly lower levels of collagen types I and III were detected in the ADM than in the control group. However, the LD group showed no significant difference between the control group or the ADM group, in the level of collagen types I and III (mRNA ratio expression of collagen type I: control group, 1.0084 ± 0.194; ADM group, 0.2418 ± 0.0463; LD flap group, 0.6265 ± 0.1708; collagen type III: control group, 1.0006 ± 0.0382; ADM group, 0.3503 ± 0.0693; LD flap group, 0.6717 ± 0.0382; Figs. 16 and 17 ).

## Discussion

Capsular contracture of breast implants is one of the most difficult complications of breast surgery. It is reported that up to 20% of implants may develop contracture within a decade, with postreconstruction cases, especially those involving radiation, exhibiting the highest rates. 14 While capsule formation is a typical response to foreign bodies, the clinical significance arises when excessive scar formation and thickened capsules cause distortion, firmness, and shrinkage. The hypertrophic scar theory proposes that myofibroblasts are stimulated in the capsule, creating a scar and contracture. 15 Based on these points, we attempted to measure the quantitative effect of ADM, which is known to have an excellent preventive effect on capsular contraction in humans. The experiment was designed to quantitatively compare autologous tissues, ADM, and a bare implant.

From the obtained peri-implant capsule, we compared the following among groups. The physical properties of capsular contracture were confirmed through capsule thickness and tensile strength, which is related to the risk of firmness and distortion. TGF-β1 and TGF-β2 associated with the synthesis and distribution of collagen and fibrosis in foreign body reactions were measured from the obtained capsule. 16 17 In this context, SMAD3—which mediates TGF-β, transmits signals through phosphorylation was quantified by RT-qPCR. 18 α-SMA, a factor associated with myofibroblasts, which is a collagen producer after transformation from fibroblasts, also plays a critical role in capsule contracture formation. 19 Immature type III collagen and mature type I collagen which both result from fibroblast activity stimulated by TGF-β, were also detected by mRNA expression.

The results of our experiment were also consistent with the hypertrophic scar theory. The myofibroblast activity was lower in the ADM and LD groups than in the control group but only the LD group showed a significant difference. TGF-β1 and SMAD3, which are important factors involved in the process of collagen synthesis and fibrosis in foreign body reactions, were also decreased in both experimental groups but a significant difference was only shown in the ADM group. In addition, as fibrosis progressed, the activity of collagen type I and III produced in fibroblasts was decreased in both experimental groups, but only the ADM group showed a marked difference. The physical properties of capsular contracture (capsule thickness and tensile strength) which are associated with peri-implant firmness and distortion were measured significantly lower in both the experimental groups than the control group.

In the comparison between the LD group and the ADM group, there was no difference. When compared with the control group and each of the LD group and the ADM group, the LD group showed significantly lower myofibroblast activity (α-SMA), and the ADM group showed markedly lower values on TGF-β1, SMAD3, and collagen types I and III. The LD group was able to prove their effectiveness on myofibroblast activity when compared with the control group. However, TGF-β1, which is an important factor involved in the process of collagen synthesis and fibrosis in foreign body reactions, was significantly decreased in the ADM group when compared with the control group. Also, the same result was shown on SMAD3 which affects TGF-β1 signaling. Furthermore, the activity of collagen types I and III produced in fibroblasts was also significantly less in the ADM group than control group. However, in a direct comparison of the prevention of capsular contracture between the LD group and the ADM group, one group did not show a superior effect compared to the other group.

Wrapping of the implant with a thick tissue such as ADM or LD muscle reduces the possibility of capsular contracture. Our study administered radiation specifically to induce this condition, allowing us to evaluate the protective efficacy of different covering materials. Especially, about ADM, the results of numerous studies highlight the preventive benefits of ADM on capsular contracture. 20 21 Recent studies have shown that ADM has a protective effect against radiation-induced capsular contracture models. Moyer et al reported that irradiated ADM showed less peri-implant inflammation and nonvascular α-smooth actin than native radiated capsules. 22 Furthermore, ADM appears to limit the elastosis and chronic inflammation seen in irradiated implant reconstructions. In their rodent model study, Komorowska-Timek et al observed a marked reduction in inflammation and suppression of pseudoepithelial formation in ADM-treated capsules after irradiation, as opposed to those without ADM coverage. 23

However, our experimental results showed no significant difference in the prevention of capsular contracture between the ADM group and the LD group. Especially, there was no significant difference in the results of physical properties (capsule thickness and tensile strength) which can reflect the degree of contracture. The ADM group had the advantage of diminishing peri-implant inflammation and preventing elastosis, and the LD group had the advantage of surrounding the implant with thicker tissue. There were concerns about muscle damage caused by irradiation while inducing capsular contracture in the LD group, but as a result, a comparison between the ADM groups showed similar results in preventing capsular contracture.

Also, the problem to consider with ADM is that it is not free from cost issues. Even though some feel burdened by the high cost of ADM, implant-based reconstruction has an advantage in shorter operation time, anesthesia time, and hospitalization period over autologous breast reconstruction. In several studies comparing the cost-effectiveness on breast reconstruction, the results show that the implant-based reconstruction group is lower or similar to autologous reconstruction. 24 25 Also, implant-based reconstruction is free from donor morbidity which takes high advantage.

In this study, we induced capsular contracture using radiation. Many studies have reported that radiotherapy exacerbates capsular contracture after breast implant reconstruction. However, capsular contracture is rarely induced by radiation in animal models. 4 26 In particular, induction of capsular contraction using radiation is even rarer in rabbits. We established the amount of radiation required to induce capsular contraction in rabbits based on our previous experiments and the opinion of a radiation oncologist. 4 The results of the experiments were further clarified by inducing capsular contracture by irradiating the animals with a predetermined amount of radiation. Our experiments may facilitate the selection of radiation doses when planning animal experiments with rabbits to induce capsular contraction using radiation.

In our radiation-induced capsular contracture model in rabbit model, we compared groups of dual-plane (control, ADM coverage, and LD coverage group). Two groups of dual-plane (ADM coverage and LD coverage group) were able to prove their effectiveness in preventing capsular contracture when compared with the control group. However, there was no significant advantage in reducing capsular contracture between groups of dual-plane. To find the most suitable method for capsular contracture protection, a comparison with prepectoral implant reconstruction, which wraps the ADM completely, may be considered in future research.

### Conclusion

The placement of ADM and LD flaps was associated with a significant reduction in fibrotic factors, leading to significantly decreased levels of collagen and myofibroblast activation relative to the control. On the expression of fibrosis marker comparison, the LD group demonstrated a markedly pronounced decrease in myofibroblast activity (α-SMA) than the control group. In the ADM group, TGF-β1 and SMAD3 levels were significantly lower, and the activity of collagen types I and III produced in fibroblasts was significantly lower than the control group. However, there was no significant difference between the ADM group and the LD group on physical properties and fibrosis markers. Therefore, it can be expected that ADM and LD have similar effects on reducing the risk of capsular contracture in a radiation-induced capsular contracture rabbit model.
